# Association analysis of *PON2 *genetic variants with serum paraoxonase activity and systemic lupus erythematosus

**DOI:** 10.1186/1471-2350-12-7

**Published:** 2011-01-11

**Authors:** Sudeshna Dasgupta, F Yesim Demirci, Amy S Dressen, Amy H Kao, Elisa Y Rhew, Rosalind Ramsey-Goldman, Susan Manzi, Candace M Kammerer, M Ilyas Kamboh

**Affiliations:** 1 Department of Human Genetics, University of Pittsburgh, Pittsburgh, PA, USA; 2 Division of Rheumatology and Clinical Immunology, Lupus Center of Excellence, Pittsburgh, PA, USA; 3 Division of Rheumatology, Feinberg School of Medicine, Northwestern University, Chicago, IL, USA; 4 West Penn Allegheny, Pittsburgh, PA, USA

## Abstract

**Background:**

Low serum paraoxonase (PON) activity is associated with the risk of coronary artery disease, diabetes and systemic lupus erythematosus (SLE). Our prior studies have shown that the *PON1*/rs662 (p.Gln192Arg), *PON1*/rs854560 (p.Leu55Met), *PON3*/rs17884563 and *PON3*/rs740264 SNPs (single nucleotide polymorphisms) significantly affect serum PON activity. Since *PON1, PON2 *and *PON3 *share high degree of structural and functional properties, in this study, we examined the role of *PON2 *genetic variation on serum PON activity, risk of SLE and SLE-related clinical manifestations in a Caucasian case-control sample.

**Methods:**

*PON2 *SNPs were selected from HapMap and SeattleSNPs databases by including at least one tagSNP from each bin defined in these resources. A total of nineteen *PON2 *SNPs were successfully genotyped in 411 SLE cases and 511 healthy controls using pyrosequencing, restriction fragment length polymorphism (RFLP) or TaqMan allelic discrimination methods.

**Results:**

Our pair-wise linkage disequilibrium (LD) analysis, using an *r*^*2 *^cutoff of 0.7, identified 14 *PON2 *tagSNPs that captured all 19 *PON2 *variants in our sample, 12 of which were not in high LD with known *PON1 *and *PON3 *SNP modifiers of PON activity. Stepwise regression analysis of PON activity, including the known modifiers, identified five *PON2 *SNPs [rs6954345 (p.Ser311Cys), rs13306702, rs987539, rs11982486, and rs4729189; *P *= 0.005 to 2.1 × 10^-6^] that were significantly associated with PON activity. We found no association of *PON2 *SNPs with SLE risk but modest associations were observed with lupus nephritis (rs11981433, rs17876205, rs17876183) and immunologic disorder (rs11981433) in SLE patients (*P *= 0.013 to 0.042).

**Conclusions:**

Our data indicate that *PON2 *genetic variants significantly affect variation in serum PON activity and have modest effects on risk of lupus nephritis and SLE-related immunologic disorder.

## Background

Systemic lupus erythematosus (SLE), a systemic autoimmune disease with high female predominance (female to male ratio = 9:1) in reproductive years (15-44 years), is characterized by a pronounced inflammation and the production of a variety of autoantibodies against multiple antigens. SLE pathogenesis is influenced by several genes, hormones and environmental agents. Familial aggregation and high heritability (up to 66%) suggest that the genetic elements have strong influence on SLE predisposition [1,2].

A prime cause of high mortality rate in SLE women is attributed to an unusually high rate of premature coronary artery disease (CAD) with risk of myocardial infarction 50 times higher than the general population [3]. The paraoxonase (*PON*) genes have received major attention as antioxidants that attenuate oxidation of low density lipoprotein (LDL), a key regulator in the pathogenesis of atherosclerosis leading to several cardiovascular diseases [4]. Low serum paraoxonase (PON) activity (using paraoxon as a substrate) is a significant risk factor for CAD [5,6]. Low PON activity is also associated with SLE risk, independent of other known risk factors [7,8]. PON activity is under genetic control and the variants in the *PON1 *gene have strong influences on PON activity variation [7,9].

*PON2 *is a positional candidate for SLE risk as the entire *PON *locus comprising three genes (*PON1, PON2 and PON3*) on chromosome 7q21.3 lies close to a linkage peak for SLE (LOD score of 2.40) in the same region (7q21-22) [10,11]. All *PON *genes are antioxidants and can hydrolyze a variety of substrates [4,12]. *PON1 *and *PON3 *are mainly expressed in the liver and their protein products circulate in the blood as HDL-bound, while *PON2 *is ubiquitously expressed in human tissues and its product remains mostly intracellular as membrane-bound [4]. *PON2 *gene spans nearly 30 kb on chromosome 7 with 9 exons encoding two protein isoforms of 354 (NCBI Reference Sequence: NP_000296.2) and 342 (NCBI Reference Sequence: NP_001018171.1) amino acids [10]. Two common nonsynonymous *PON2 *coding variants have been reported to date [rs11545941 (p.Ala148Gly) and rs6954345 (p.Ser311Cys)] and the latter has been hypothesized to be a catalytic center for the hydrolysis of oxidized lipids [13]. So far, the *PON2 *reports mainly examined these two coding polymorphisms, of which the rs6954345 (p.Ser311Cys) SNP was found to be associated with several cardiovascular complications [14-16] and renal dysfunction [17], whereas the rs11545941 (p.Ala148Gly) SNP associated with plasma lipoprotein levels in some studies [18]. Recently, the PON2 protein was reported to degrade and inactivate *N*-3-oxododecanoyl-homoserine lactone, the quorum sensing signal of pathogenic bacteria [19] but its exact physiologic or pathophysiologic role has yet to be identified. PON2 protein shows highest lactonase activity among all PON proteins and the rs6954345 (p.Ser311Cys) SNP in recombinant *PON*2 was recently shown to cause altered glycosylation and impaired lactonase activity [20].

Our previous reports with SLE datasets have identified low PON activity as an independent risk factor for SLE and our analysis of *PON1 *and *PON3 *SNPs revealed some modest associations with lupus nephritis [7,21]. The *PON1 *and *PON3 *SNPs account for 30-40% of serum PON activity variation in Caucasians [7,21]. Given that *PON1-2-3 *gene cluster resides close to a linkage peak for SLE and our previous observations suggesting a relationship between SLE and PON activity/*PON1-3 *genetic variation, the purpose of this study was to investigate the remaining member of the *PON *multigene family, *PON2*. We genotyped and analyzed 19 highly informative *PON2 *SNPs selected from HapMap and SeattleSNPs databases in a Caucasian SLE case-control sample. To our knowledge, this is the first comprehensive association study of the *PON2 *variants with risk of SLE, SLE-related clinical features and serum PON activity.

## Methods

### Subjects

A total of 411 Caucasian women with SLE and 511 Caucasian healthy women with no apparent history of SLE were studied. Peripheral blood samples were collected at the baseline visit and used for genotyping and measurement of serum PON activity (at the Pittsburgh site only) as described previously [7].

Of 411 SLE cases, 344 belonged to the Pittsburgh Lupus Registry and the rest (n = 67) to the Chicago SOLVABLE study (Study of Lupus Vascular and Bone Longterm Endpoints). All controls enrolled in our study were geographically matched to the cases: 454 controls were recruited from the Central Blood Bank of Pittsburgh and the rest (n = 57) from the Chicago SOLVABLE study. Demographic and clinical characteristics of SLE patients and controls are presented in Table 1 and described in detail elsewhere [22,23]. All SLE subjects were adult women aged 18 years and older and met the 1982 [24] and 1997 [25] revised American College of Rheumatology (ACR) classification criteria for SLE. The subset of the ACR criteria that were included in association analysis are shown in Table 1 [highly frequent (i.e. Arthritis and ANA) or infrequent (i.e. Neurologic disorder) subphenotypes were not suitable for a meaningful analysis given the size of our sample]. Also included in Table 1 is the percentage of SLE cases who had experienced one or more of physician-confirmed cardiac and vascular events (myocardial infarction, coronary artery bypass graft surgery, percutaneous transluminal coronary angioplasty, angina pectoris, cardiac death, stroke, transient ischemic attack, congestive heart failure, blood clots, or vascular surgery) in a subset of the SLE sample in which this data was available (n = 292). The mean ± standard deviation (SD) age was 43.5 ± 11.4 years for cases and 45.7 ± 12.9 years for controls. This study was approved by the institutional review board and all subjects gave written, informed consent.

**Table 1:** Characteristics of the study population

	Cases (n = 411)	Controls (n = 511)	Combined (n = 922)
Mean age	43.54 ± 11.36	45.68 ± 12.86	44.68 ± 12.22
Body mass index (kg/m^2^)	26.90 ± 6.82	27.34 ± 6.59	27.14 ± 6.70
Current and/or Former Smoker (%)	37.84%	40.29%	39.23%
PON activity (in units/liter)*	613.88 ± 412.94	709.85 ± 496.39	671.44 ± 466.87
SLE manifestations (%)**			
Skin rash (malar and/or discoid)	55%	-	-
Photosensitivity	63%	-	-
Oral ulcers	54%	-	-
Serositis (pleuritis or pericarditis)	44%	-	-
Renal involvement	30%	-	-
Hematologic involvement***	54%	-	-
Immunologic involvement****	72%	-	-
Cardiac and vascular events*****	33%	-	-

*Available only in a subset of the sample (291 cases and 436 controls)

** The subset of American College of Rheumatology (ACR) classification criteria for SLE that were included in association analysis

*** Hemolytic anemia or leukopenia (on two or more occasions) or lymphopenia (on two or more occasions) or thrombocytopenia

**** Anti-DNA antibody or anti-Sm antibody or positive finding of antiphospholipid antibodies

*****Data available only in a subset of SLE patients (292 cases)

### Selection of PON2 SNPs and genotyping

**Figure 1:**
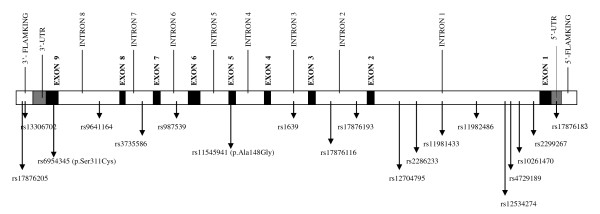
**Locations of the genotyped 19 *PON2 *SNPs**. The exons are depicted as black bars (UTRs are highlighted as gray) and introns & flanking regions as white bars.

We initially selected a total of twenty highly informative *PON2 *SNPs [residing within *PON2 *or 1-kb flanking regions and with ≥ 4% minor allele frequency (MAF)], by using the LD and tagSNP information available for Caucasians in the International HapMap http://www.hapmap.org and SeattleSNPs http://pga.gs.washington.edu/ databases (Figure 1). The tagSNP bins identified in these 2 databases were not identical (likely due to the sampling and methodological differences), thus we selected at least one tagSNP from each bin defined in these resources. One SNP (*PON2*/rs17876195) could not be genotyped due to technical difficulties, therefore a total of 19 successfully genotyped *PON2 *SNPs were included in our analyses.

Genomic DNA was isolated from the buffy coat using the QIAamp DNA kits (QIAGEN, Chatsworth, CA). Ten *PON2 *SNPs (rs11982486, rs2286233, rs17876116, rs11545941 (p.Ala148Gly), rs17876193, rs3735586, rs10261470, rs9641164, rs13306702 and rs17876183) were genotyped by Pyrosequencing using the PSQ HS 96 system (Biotage, Uppsala, Sweden) and the corresponding PCR and sequencing primers were provided in the Additional File 1, Table S1.

One of the *PON2 *coding SNPs, *PON2*/rs6954345 (p.Ser311Cys), was genotyped by restriction fragment length polymorphism (RFLP) analysis using the restriction enzyme *DdeI*. The PCR primers (forward = 5'-AACAGGGCTTATTGATGATTGAGT-3' and reverse = 5'-ACAGACCCATTGTTGGCATAA-3') amplified a 145 bp product containing the SNP of interest. *Dde*I digestion of this product yielded a single fragment of 145 bp for GG genotype (CysCys), 2 fragments of 100 and 45 bp for CC genotype (SerSer), and 3 fragments of 145, 100 and 45 bp for the heterozygous CG genotype (SerCys).

The remaining eight *PON2 *SNPs (rs2299267, rs12534274, rs987539, rs4729189, rs11981433, rs12704795, rs1639 and rs17876205) were genotyped by TaqMan allelic discrimination on an ABI 7900HT instrument (Applied Biosystems, Foster City, CA) using the following SNP genotyping assays: C___2630173_1_ for *PON2/*rs2299267, C__31373224_10 for *PON2/*rs12534274, C___8952813_10 for *PON2/*rs987539, C__27922117_10 for *PON2/*rs4729189, C___2630169_10 for *PON2/*rs11981433, C__26570646_10 for *PON2/*rs12704795, C__11708890_10 for *PON2/*rs1639, and C__59001801_10 for *PON2/*rs17876205.

### Statistical analysis

Allele frequencies were calculated by allele counting and all SNPs were tested for deviations from Hardy-Weinberg Equilibrium (HWE) expectations. Haploview http://www.broadinstitute.org/mpg/haploview was used to assess the pairwise LD between all *PON2 *SNPs as well as the two *PON1 *SNPs [*PON1*/rs662 (p.Gln192Arg) and *PON1*/rs854560 (p.Leu55Met)] and the two *PON3 *SNPs (*PON3*/rs740264 and *PON3*/rs17884563) that we previously reported to influence PON activity.

Data on PON activity was available only at the Pittsburgh site and the raw measurements were square root transformed to reduce non-normality. We conducted combined (forward and reverse) stepwise and multiple regression analyses to test for independent association of PON activity with environmental covariates [disease status, age, body mass index (BMI), smoking] [7] as well as the two *PON1 *SNPs [*PON1*/rs662 (p.Gln192Arg) and *PON1*/rs854560 (p.Leu55Met)], one *PON3 *SNP (*PON3*/rs17884563) and 13 *PON2 *SNPs that were in low LD. Allele effects were modeled as additive for common SNPs and as dominant for relatively uncommon variants. To determine whether additional, unmeasured genetic variation in *PON2 *might also influence PON activity, we also evaluated the association of *PON2 *haplotypes with PON activity.

Association of SLE status with *PON2 *SNPs was evaluated using logistic regression and the recruitment site (Pittsburgh or Chicago) and age (found to be significant in stepwise regression analysis of potential covariates examined, including the recruitment site, age, BMI and smoking) were included as covariates in the model. SNP effects were modeled as additive for common variants and dominant for relatively uncommon ones. Within SLE cases, the logistic regression analysis was performed to test for association between *PON2 *SNPs and SLE-related subphenotypes [skin involvement (malar rash and/or discoid rash), lupus nephritis, photosensitivity, oral ulcers, serositis, immunologic disorder and hematologic involvement] using a dominant model and including recruitment site and age as covariates.

All regression and haplotype analyses were performed using the R Statistical Suite (R Foundation for Statistical Computing, Vienna, Austria). Power analysis was performed using the Quanto program http://hydra.usc.edu/GxE.

## Results

### Pair-wise LD analysis of PON2 SNPs and relevant PON1 and PON3 SNPs

**Figure 2:**
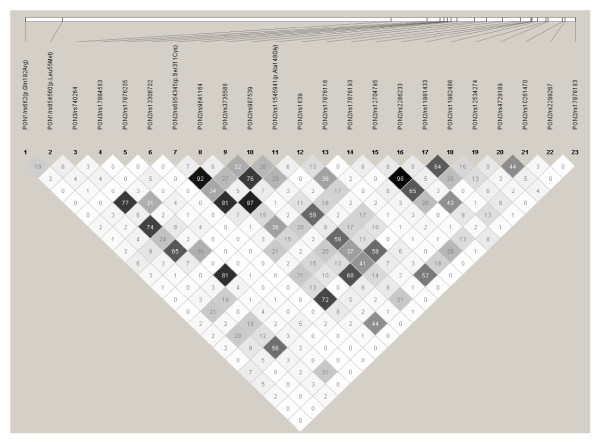
**Pairwise linkage disequilibrium plot of *PON1*/rs662 (p.Gln192Arg), *PON1*/rs854560 (p.Leu55Met), *PON3*/rs740264, *PON3*/rs17884563, and 19 *PON2 *SNPs in the combined sample (cases & controls) showing *r***^***2***^** (×100) values**.

For all 19 *PON2 *SNPs, the observed minor allele frequencies (MAFs) in our control sample were similar to those reported in the HapMap database for Caucasians. The observed genotype frequencies of all *PON2 *SNPs were in accordance with HWE. Pairwise LD analyses revealed similar patterns in cases and controls, hence LD was computed by combining these two groups (Figure 2). Significant correlations (*r*^*2 *^≥ 0.7) were found between five *PON2 *SNP pairs [*PON2*/rs6954345 (p.Ser311Cys) & rs11545941 (p.Ala148Gly), *r*^*2 *^= 0.81)], [*PON2*/rs6954345 (p.Ser311Cys) & rs12534274, *r*^*2 *^= 0.72], [*PON2*/rs6954345 (p.Ser311Cys) & rs3735586, *r*^*2 *^= 0.92], [rs1639 & rs9641164; *r*^*2 *^= 0.87] and [rs11981433 & rs12704795; *r*^*2 *^= 0.98]. Hence, one of the SNPs from each of these 5 highly correlated SNP pairs [rs11545941 (p.Ala148Gly), rs12534274, rs3735586, rs1639, and rs12704795] were excluded from subsequent analyses. Although all 19 *PON2 *SNPs were reported as tagSNPs in at least one of the two publicly available databases using different *r*^*2 *^cutoffs (HapMap and SeattleSNPs), a total of 14 tagSNP bins were identified in our sample using an *r*^*2 *^cutoff of 0.7.

Because *PON1*, *PON2 *and *PON3 *genes lie adjacent to each other and *PON1 *and *PON3 *variants are known modifiers of PON activity, we also assessed LD between the *PON2 *variants and the four *PON1 *and *PON3 *SNPs that were previously found to be associated with PON activity (Figure 2). Significant correlations (*r*^*2 *^≥ 0.7) were observed between *PON2*/rs6954345 (p.Ser311Cys) and *PON3*/rs740264 (*r*^*2 *^= 0.77) and between *PON2*/rs17876193 and *PON3/*rs17884563 (*r*^*2 *^= 0.81). From the first correlated SNP pair, *PON2*/rs6954345 (p.Ser311Cys) was included in subsequent analysis for PON activity (regression and haplotype) since this SNP had also tagged three other *PON2 *SNPs [rs11545941 (p.Ala148Gly), rs12534274 & rs3735586]. From the second correlated SNP pair, *PON3*/rs17884563 was included in subsequent analysis of PON activity.

### PON2 tagSNPs and serum PON activity

Table 2 shows the *P *and R^2 ^values from stepwise linear regression analysis of PON activity with *PON2 *tagSNPs along with the significant PON activity modifiers from *PON1 *[7] and *PON3 *[21] genes and possible environmental covariates (disease status, age, BMI, and smoking status). In addition to previously reported effects of disease status, age, and the *PON1 *and *PON3 *SNPs, we identified associations with 5 *PON2 *SNPs: rs13306702 (*P *= 0.005), *PON2*/rs6954345 (p.Ser311Cys) (*P *= 2.1 × 10^-6^), rs987539 (*P *= 3.4 × 10^-5^), rs11982486 (*P *= 7.0 × 10^-4^), and rs4729189 (*P *= 0.003). The same environmental covariates and SNPs were significant when all covariates and SNPs were entered in the model simultaneously. The beta values listed in Table 2 represent the effect of the minor allele of each of 5 *PON2 *SNPs on PON activity (square root transformed). PON activity increased with the presence of minor alleles of *PON2*/rs6954345 (p.Ser311Cys) and *PON2*/rs13306702 SNPs, and decreased with the presence of the minor alleles of the remaining 3 significant *PON2 *SNPs. Altogether, these 5 *PON2 *SNPs explained 3.4% of the variation in PON activity.

**Table 2:** Significant results obtained from stepwise linear regression analysis of serum PON activity (square root transformed) with SLE disease status, age, BMI, smoking, *PON1*/rs662 (p.Gln192Arg), *PON1*/rs854560 (p.Leu55Met) and *PON3*/rs17884563 SNPs and 13 *PON2 *tagSNPs

FACTOR	MAF	*Beta*	*P*	***R***^***2***^
disease		-2.22	3.8 × 10^-8^	0.015
age		0.04	0.013	0.003
*PON1*/rs662 (p.Gln192Arg)	0.280	10.54	<2.0 × 10^-16^	0.392
*PON1*/rs854560 (p.Leu55Met)	0.358	-1.96	1.1 × 10^-8^	0.017
*PON3*/rs17884563	0.092	-1.03	0.082	0.001
*PON2*/rs13306702	0.017	3.87	0.005	0.004
*PON2*/rs6954345 (p.Ser311Cys)	0.233	2.22	2.1 × 10^-6^	0.011
*PON2*/rs987539	0.454	-3.64	3.4 × 10^-5^	0.009
*PON2*/rs11982486	0.330	-2.84	7.0 × 10^-4^	0.006
*PON2*/rs4729189	0.223	-2.61	0.003	0.004

To assess whether unmeasured variants might influence PON activity, we also performed haplotype analysis for PON activity (Table 3). Because of the strong effects of *PON1 *and *PON3 *SNPs on PON activity, we performed haplotype analysis after adjusting for disease status, age, BMI, smoking, *PON1*/rs662 (p.Gln192Arg), *PON1*/rs854560 (p.Leu55Met), and *PON3*/rs17884563. Only one haplotype, Hap 6, was significantly associated with increased PON activity (*P *= 6.1 × 10^-5^) compared to the most common haplotype (Hap Base). This haplotype contained the minor allele for *PON2*/rs6954345 (p.Ser311Cys) that is the most significant of the *PON2 *variants. Therefore, this result is consistent with our stepwise regression analysis of *PON2 *genotypes and indicates that no specific haplotype influences PON activity independent of the analyzed *PON2 *genotypes.

**Table 3:** Haplotype analysis of 13 *PON2 *tagSNPs in relation to PON activity adjusted for disease status, age, BMI, smoking, *PON1*/rs662 (p.Gln192Arg), *PON1*/rs854560 (p.Leu55Met), and *PON3*/rs17884563

	Haplotypes							
	
	Hap 1	Hap 2	Hap 3	Hap 4	Hap 5	Hap 6	Hap Rare	Hap Base
***PON2/*rs17876205**	G	G	G	G	G	G	*	G
***PON2/*rs13306702**	G	G	G	G	G	G	*	G
***PON2/*rs6954345 (p.Ser311Cys)**	C	C	C	C	C	G	*	C
***PON2/*rs9641164**	A	A	A	T	T	A	*	A
***PON2/*rs987539**	C	C	C	T	T	T	*	C
***PON2/*rs17876116**	G	G	G	G	G	G	*	G
***PON2/*rs2286233**	A	T	T	A	A	A	*	A
***PON2/*rs11981433**	C	T	T	T	T	T	*	C
***PON2/*rs11982486**	T	T	T	T	T	T	*	C
***PON2/*rs4729189**	T	T	T	A	A	A	*	A
***PON2/*rs10261470**	A	A	G	G	G	G	*	G
***PON2/*rs2299267**	A	A	A	A	G	A	*	A
***PON2/*rs17876183**	G	G	G	G	G	G	*	G
**Haplotype Frequency**	0.073	0.051	0.052	0.052	0.096	0.192	0.161	0.323
**Coefficient**	0.21	1.14	0.31	0.47	-0.57	1.66	0.06	
**Standard Error**	0.56	0.72	0.68	0.70	0.80	0.41	0.44	
**T statistic**	0.38	1.59	0.46	0.67	-0.72	4.04	0.14	
***P***	0.705	0.113	0.643	0.506	0.475	6.1 × 10^-5^	0.890	

### PON2 tagSNPs and SLE risk

We examined the relation of 14 *PON2 *tagSNPs with SLE risk through single-site and haplotype analyses. Four *PON2 *tagSNPs had less than 5 individuals who were homozygous for the minor allelle: *PON2*/rs13306702, *PON2*/rs17876116, *PON2*/rs17876183 and *PON2*/rs17876205. For these SNPs, the rare homozygotes were combined with the heterozygote individuals for the association analysis with SLE risk (dominant model). The remaining SNPs (MAF range: ~10-45%) were analyzed under the additive model for which we had 80% power to detect a minimum OR of ~1.5-1.3 at the 5% significance level. In single-site analysis, the genotype frequencies of all *PON2 *tagSNPs were comparable between cases and controls. Like single-site, no significant association was found with the 14-site *PON2 *haplotype analysis (data not shown).

### PON2 tagSNPs and SLE-related clinical manifestations

In addition to SLE risk, we examined the association of 14 *PON2 *tagSNPs with seven SLE-related subphenotypes within the SLE sample, by stratifying the SLE cases for the presence and absence of the subphenotype of interest and using a dominant model. Three *PON2 *tagSNPs (rs11981433, rs17876183, and rs17876205) showed modest association with lupus nephritis (age and recruitment site adjusted *p*-values ranging between 0.013-0.032). The genotype distributions of these SNPs in SLE cases with/without nephritis and the age and recruitment site adjusted ORs were as follows; for rs11981433 TT: 43.80%/33.68% & TC+CC: 56.20%/66.32% (OR: 0.61, 95% CI: 0.39-0.96, *P *= 0.032), for rs17876183 GG: 93.50%/98.25% & GA+AA: 6.50%/1.75% (OR: 4.63, 95% CI: 1.37-15.59, *P *= 0.013), and for rs17876205 GG: 93.50%/98.24% & GC+CC: 6.50%/1.76% (OR: 4.09, 95% CI: 1.26-13.24, *P *= 0.019). One of these SNPs, rs11981433, showed also modest association with immunologic disorder in the same direction as observed for lupus nephritis (TT: 39.66%/29.31% & TC+CC: 60.34%/70.69%, adjusted OR: 0.62, 95% CI: 0.39-0.98, *P *= 0.042). No other significant associations were observed between *PON2 *tagSNPs and SLE-related subphenotypes evaluated in this study.

## Discussion

Inter-individual variation in serum PON activity is under strong genetic influence and *PON1 *genetic variants appear to be the main contributors [7,9]. We have previously shown that in addition to *PON1 *SNPs, the *PON3 *SNPs also influence serum PON activity, although at a lesser degree [7,21]. Altogether *PON1*/rs662 (p.Gln192Arg), *PON1*/rs854560 (p.Leu55Met), *PON3*/rs740264 and *PON3*/rs17884563 SNPs account for more than 40% of variation in serum PON activity among Caucasians [7,21]. Since the impact of *PON2 *SNPs on PON activity has not been comprehensively evaluated to date, we examined the role of *PON2 *tagSNPs in regulation of serum PON activity while taking also into account the effects of the SNPs in *PON1 *and *PON3 *genes that lie in close vicinity of *PON2*.

We found significant associations of one coding and four non-coding *PON2 *tagSNPs, [rs6954345 (p.Ser311Cys), rs13306702, rs987539, rs11982486, and rs4729189 with *p-*values ranging from 0.005 to 2.1 × 10^-6^] with PON activity, after incorporating effects of relevant *PON1 *and *PON3 *SNPs as well as environmental covariates. The overall contribution of *PON2 *genetic variation on PON activity was 3.4%. A similar association of the *PON2*/rs6954345 (p.Ser311Cys) SNP with serum PON activity in the same direction (minor allele associated with increased PON activity) has also been reported by Mackness et al. (2000) in type II diabetic individuals [26]. Although *PON2*/rs6954345 (p.Ser311Cys) SNP is highly correlated with *PON3*/rs740264 SNP which is a known modifier of PON activity (*r*^*2*^*=*0.77; see the second paragraph in LD analysis results), the observation of 4 additional independently and highly significantly associated *PON2 *tagSNPs supports a possible independent effect of *PON2 *genetic variation on PON activity.

The lower contribution of *PON2 *on PON activity as compared to *PON1 *and *PON3 *is likely due to the fact that unlike PON1 and PON3 proteins that are bound to circulating HDL in the blood, PON2 protein is believed to be predominantly intracellular and membrane bound [27-30]. The observed effect on PON activity may be related to a low level secretion of PON2 enzyme into blood [27] and/or the presence of a low-abundant secreted PON2 isoform. Ng et al. (2001) [27] failed to detect PON2 protein secretion by Western analysis despite the presence of a "putative consensus signal sequence" in PON2 N-terminal region (similar to that in PON1 and PON3), therefore they argued that the protein could either be subject to rapid degradation following its secretion or secreted at a very low level. Although most studies mainly focused on two PON2 isoforms [29,31], Mochizuki et al. (1998) [32] reported that alternative splicing of *PON2 *transcript results in several *PON2 *splice forms. Nevertheless, our results indicate only a small effect of *PON2 *variation on serum PON activity and confirms that the circulatory HDL-bound PON1 is the major modulator. Lack of complete knowledge regarding the natural substrate for PON2 enzyme is a major limiting factor in evaluating the role of *PON2 *genetic variation in PON2 enzymatic activities.

Although several studies linked *PON2 *variants to various diseases like amyotrophic lateral sclerosis [33,34], Alzheimer's disease [35], CHD [14-16], and metabolic traits [18] with controversial findings [36], there is no published study that links *PON2 *with SLE risk. Regardless of the association observed between *PON2 *variants and PON activity, we did not find any major contribution of *PON2 *genetic variation to overall SLE risk neither in single-site or haplotype analyses. This is consistent with our prior observations regarding the relation between *PON1 *and *PON3 *SNPs and SLE risk [7,21].

Lupus nephritis is a major cause of mortality in SLE patients [1]. Some studies reported associations of *PON2 *genetic variation with renal dysfunction or nephropathy [17,37]. Our group previously reported modest associations of three *PON1 *promoter SNPs with lupus nephritis in Caucasian SLE patients [7]. Similarly, we observed modest associations of *PON2 *SNPs with lupus nephritis in Caucasian SLE cases (rs11981433, rs17876183 and 17876205 with covariate-adjusted *p*-values ranging between 0.013-0.032). Our pairwise LD analysis of the nephritis-associated three *PON1 *promoter SNPs [7] (rs705379, rs705381 and rs854573) and three *PON2 *SNPs did not reveal high correlations between these SNPs (data not shown). A large prospective study [17] comprising 3,374 diabetic subjects evaluated *PON2 *variations with indices of renal dysfunction and reported the association of *PON2*/rs6954345 (p.Ser311Cys) and rs12704795 SNPs with renal dysfunction. Although no association was observed with *PON2*/rs6954345 (p.Ser311Cys) SNP and lupus nephritis in our study, significant association was found with *PON2*/rs11981433 SNP that is highly correlated with *PON2*/rs12704795 SNP (*r*^*2 *^= 0.98). Our results also revealed a modest association of *PON2*/rs11981433 SNP with immunologic disorder (adjusted *P *= 0.042). Although the associations observed with SLE subphenotypes were not strong enough to survive correction for multiple testing, the association of rs11981433 SNP with more than one SLE subphenotype in the same direction (protective) would argue in favor of its possible genuine effect on SLE-related clinical features.

## Conclusions

In summary, our results suggest that *PON2 *genetic variation is significantly and independently associated with variation in serum PON activity (after incorporating effects of age, SLE status, relevant *PON1 *and *PON3 *variation), although to a much lesser extent than *PON1*. We found no obvious association between *PON2 *tagSNPs and SLE risk, however, we observed few modest associations with lupus nephritis and SLE-related immunological disorder which await replication in larger independent samples. A comprehensive resequencing and association analysis of *PON *gene cluster may help to better understand extent to which this locus contributes to the regulation of PON activity and to the risk of SLE-related clinical features.

## Competing interests

The authors declare that they have no competing interests.

## Authors' contributions

Author's Contributions were as follows: study conception and design (MIK), acquisition of clinical data (AHK, EYR, RRG, SM), acquisition of PON2 genetic data (SD), assistance for acquisition of genetic data (FYD, MIK), molecular and/or statistical analysis and interpretation of the data (SD, FYD, ASD, CMK, MIK), drafting of the manuscript (SD, FYD, CMK, MIK), critical revision and final approval of the manuscript (all authors).

## Pre-publication history

The pre-publication history for this paper can be accessed here:

http://www.biomedcentral.com/1471-2350/12/7/prepub

## Supplementary Material

Additional file 1 **Table S1**. PCR and Sequencing primers used for *PON2 *SNPs genotyped by Pyrosequencing. Click here for file
