# Supplementary material for: Association analysis of PON2 genetic variants with serum paraoxonase activity and systemic lupus erythematosus
Source: BMC Med Genet. 2011 Jan 11;12:7. doi: 10.1186/1471-2350-12-7 (PMC3030528; doi:10.1186/1471-2350-12-7)
Supplement: Additional file 1 — Table S1. PCR and Sequencing primers used for PON2 SNPs genotyped by Pyrosequencing. [file 1471-2350-12-7-S1.DOC]

**Additional File 1, Table S1**

PCR and Sequencing primers used for *PON2* SNPs genotyped by Pyrosequencing

| **SNPs** | **Forward PCR primer** | **Reverse PCR primer** | **Pyrosequencing primer** |
| --- | --- | --- | --- |
| *PON2*/rs11982486 | 5´-TCCTTTGACCACCCACAATTATC-3´ | 5´-CCAAACCTCAGCATCAGACAATAT-3´ | 5´-AGATTACTACCACATTAGGC-3´ |
| *PON2*/rs2286233 | 5´-TGAGGCTTACAGTCATTTTTCACG-3´ | 5´-GTTGTGGGAAAAGAGTTCCAGAT-3´ | 5´-AGTTCCAGATGTAGAACCA-3´ |
| *PON2*/rs17876116 | 5´-TTGACTGCTCCTGACATAATCACA-3´ | 5´-GCCACTACTGCAGGAAGGTTTTA-3´ | 5´-GCTCCTGACATAATCACA-3´ |
| *PON2*/rs11545941 (p.Ala148Gly) | 5´-AACCACCCAGAATTCAAGAATACA- 3´ | 5´-TGACTGTTTTCAGATGCAACAGAG-3´ | 5´-GGAAATTTTTAAATTTGAAG-3´ |
| *PON2*/rs17876193 | 5´CCAACAGAAATAACCCCAAAGA-3’ | 5´-TGTTTGCAAATGCACTGAAACTA-3´ | 5´-AAATAACCCCAAAGATAAA-3´ |
| *PON2*/rs3735586 | 5´-GGCAGGAAGGTTACCTCTAAATT-3´ | 5´-CACCAGTGTATCCAGCTCAAGTA-3´ | 5´-AAGTTTCTCATGTCATTTAG-3´ |
| *PON2/*rs10261470 | 5´-GATATGTGGAGCCCCAAATG-3´ | 5´-CACCACCTACCCCAACATTCT-3´ | 5´-GGAGCCCCAAATGGGCTG-3´ |
| *PON2*/rs9641164 | 5-´ATGCATGTACGGTGGTCTTATATT-3´ | 5´-AATGTTCTGGATGCGGAGA-3´ | 5´-TTCAGGGGATACAAAGT-3´ |
| *PON2*/rs13306702 | 5´-GGCCATATTAATTTCTCTTGTGGA-3´ | 5´-TGGGAATTTGAGTTGCAATATTT-3´ | 5´-GGAAAGCTGAAAGTGAAT-3´ |
| *PON2/*rs17876183 | 5´-CACCATCGAGCCGGGAAG-3´ | 5´ ATCCCCAGCAAGCCCACAG-3´ | 5´-ACCAGCCGCCCCATG-3´ |
